# Strength-based steeling effects in cascades of parenting adversity, children’s emotion processing, and psychological problems

**DOI:** 10.1017/S0954579425100990

**Published:** 2025-12-10

**Authors:** Patrick T. Davies, Vanessa T. Cao, Zhi Li, Meera D. Patel, Catherine Waye, Brandon Gibb

**Affiliations:** 1 Department of Psychology, University of Rochester, Rochester, NY, USA; 2 Mt. Hope Family Center, University of Rochester, Rochester, NY, USA; 3 Department of Psychology, University of Miami, Coral Gables, FL, USA; 4 Department of Psychology, Binghamton University, Binghamton, NY, USA

**Keywords:** child effortful control, child emotion knowledge, child psychopathology, family adversity, steeling effects

## Abstract

Guided by steeling and hormesis models, this paper examined parenting adversity as a quadratic predictor of children’s emotion knowledge and effortful control and, in turn, their internalizing and externalizing symptoms. Participants were 238 mothers, partners, and their preschool children (M_age_ = 4.38 years; 52% female). Multiple methods (i.e., observations, interviews, surveys, q-sorts) and informants (i.e., trained observers, experimenters, mothers, children, teachers) were used in a longitudinal design with three annual measurement occasions. Supporting the first link in the mediational cascade, lagged, autoregressive analyses indicated that a quadratic composite of parenting adversity derived from trained observer ratings of parenting at Wave 1 was a significant predictor of children’s emotion knowledge and effortful control at Wave 2. In the second part of the proposed cascade, children’s Wave 2 emotion knowledge predicted lower levels of their Wave 3 internalizing symptoms, while their Wave 2 effortful control predicted lower levels of their Wave 3 externalizing symptoms. Consistent with steeling effects, curvilinear findings in the first part of the cascade indicated that moderate levels of exposure to parenting adversity predicted the highest levels of children’s subsequent emotion knowledge and effortful control. Children also exhibited substantially diminished emotion knowledge and effortful control as their exposure to family adversity increased from moderate to high levels.

## Introduction

Children exposed to parenting adversity characterized by caregiver unresponsiveness, emotional detachment, and intrusiveness are at risk for exhibiting internalizing and externalizing symptoms (Pinquart, 2016; 2017). According to the risky families model (Repetti et al., 2002; 2011), parenting adversity increases children’s risk for psychopathology by undermining their emotion processing capacities. These emotion processing difficulties are proposed to be reflected in children’s diminished (a) effortful control, characterized by the ability to inhibit reflexive impulses to stimuli in favor of deliberate responses tailored to contextual demands (Nigg, 2017), and (b) emotion knowledge, defined as the ability to identify the nature, meaning, and origins of emotional expressions and experiences (Repetti et al., 2011; Strand et al., 2016). Research has provided some support for these mediational pathways (e.g., Cunningham et al., 2009; Hofer et al., 2010; Sadri & Yates, 2025). However, linear associations between parenting difficulties and children’s effortful control and emotion understanding are modest in magnitude and, in many cases, inconsistent (Choe et al., 2013; Eisenberg et al., 2010; Thompson et al., 2020).

In addressing the question of why parenting adversity does not robustly predict children’s emotion processing impairments in a dose–response relationship, steeling effects models in psychology have proposed that family adversity may operate as a curvilinear precursor of children’s psychological adaptation and functioning (e.g., Liu, 2015; Lemerise & Arsenio, 2000; Repetti & Robles, 2016; Rutter, 2012). Likewise, the hormesis framework, which is derived from other disciplines and recently been applied to psychology, posits that enhanced functioning conferred by moderate adversity stems from flexible, adaptive, and multi-level resiliency processes (Oshri et al., 2024; Oshri, 2023). According to steeling and hormesis models, exposure to moderate adversity in rearing contexts strengthens children’s psychological functioning by enhancing their emotion processing and regulatory capacities relative to minimal and high levels of adversity. However, research testing the viability of steeling and hormesis frameworks has, to our knowledge, focused exclusively on children’s reactivity to interparental conflict or their psychopathology symptoms (Davies et al., 2022; Hidalgo et al., 2023; Meunier et al., 2012; Oshri et al., 2024). Thus, little is known about whether the strength and nature of the mediational pathways between family adversity, children’s emotion knowledge and effortful control, and their psychological symptoms vary across levels of family adversity. To address this gap, the current study tests the steeling hypothesis that parenting adversity increases children’s risk for internalizing and externalizing symptoms through its concave relationship with emotion understanding and effortful control.

### Steeling and hormesis models of family adversity

**Figure 1.**
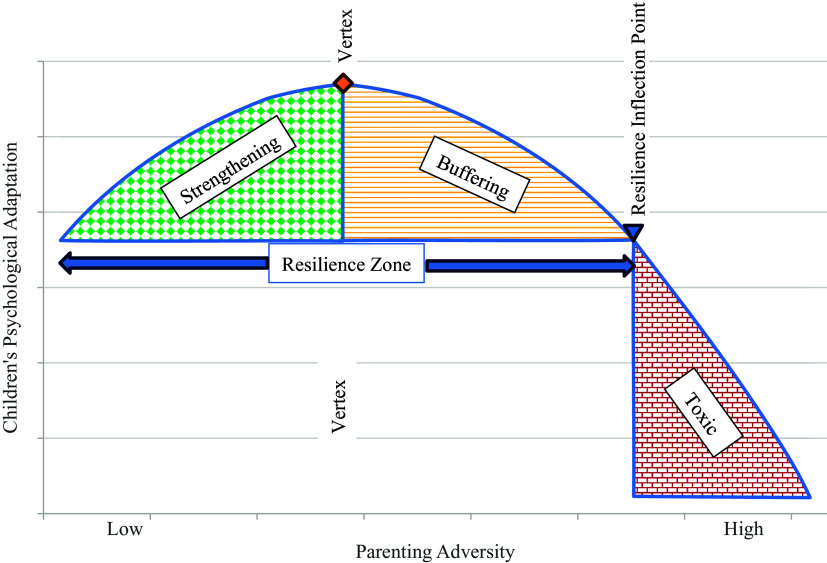
Illustration of a hormesis model of exposure to parenting adversity.

According to steeling and hormesis models (Oshri, 2023; Repetti & Robles, 2016; Rutter, 2012), experiencing psychosocial adversity may enhance children’s adaptation and inoculate them against subsequent psychological vulnerability when doses of stressors are manageable in frequency (i.e., occasional), intensity (i.e., mild), and duration (i.e., brief). Hormesis conceptualizations are particularly valuable in more precisely identifying two zones of resilience in the face of adversity (Oshri et al., 2024; Oshri, 2023). In the strengthening zone of hormesis effects illustrated in Figure 1 (i.e., green diamond matrix pattern), children’s adaptive social and emotional skills and, in turn, their mental health is reinforced as exposure to family adversity increases from negligible to mild levels (Liu, 2015; Oshri, 2023; Rutter, 2012). As the benefits of stressful experiences reach a zenith or vertex at the bend in the inverted J (see Figure 1), the advantages of experiencing adversity begin to decrease progressively. However, children are protected in this buffering region of the inverted J-shaped curve (i.e., area with yellow horizontal lines in Figure 1) because they still evidence better psychological adaptation than their peers who experience negligible adversity. The final shift of the curvilinear relationship in hormesis formulation depicts the point at which adversity becomes toxic in comparison to experiencing negligible or minimal adversity (i.e., “resilience inflection point” in Figure 1). As denoted by the “toxic” region (i.e., denoted by red bricks) of Figure 1, children exhibit progressively poorer psychological adjustment as their exposure to adversity increases from moderate to high levels. Thus, intense, prolonged, and frequent adversity is hypothesized to increase children’s vulnerability by depleting their regulatory and processing capacities (e.g., Davies & Martin, 2014).

Although the mechanisms shaping steeling or hormesis effects in adverse contexts have yet to be delineated (Oshri, 2023), there are conceptual bases for expecting that strengthening and buffering processes are manifested in emotion knowledge and regulation abilities. Steeling effects models propose that experiencing mild, intermittent adversity promotes moderate levels of emotional arousal and maximizes children’s abilities to integrate and process affective cues in social contexts (Repetti & Robles, 2016). Deeper and broader emotion processing, in turn, may promote better emotion understanding and effortful control (Lemerise & Arsenio, 2000). Moreover, mild to moderate adversity is posited to increase effortful control by facilitating the formulation, enactment, and refinement of effective coping strategies in the face of subsequent challenges (Arbel et al., 2020; Davies et al, 2022; Seery, 2011). Resulting gains in perceived coping efficacy and mastery may feedback to further promote self-regulation abilities in a transactional cycle (Repetti & Robles, 2016).

### Steeling or hormesis effects in the context of developmental psychopathology cascades

Integrating steeling effects and developmental psychopathology cascade models generates additional questions about how the proposed strengthening and buffering processes for emotion understanding and effortful control might diminish children’s risk for psychopathology in the wake of family adversity (Oshri, 2023; Repetti et al., 2011; Repetti & Robles, 2016). Effortful control and emotion knowledge may reduce children’s risk for internalizing and externalizing symptoms through common classes of mechanisms. For example, effortful control may protect children against psychopathology by downregulating distress and anger (i.e., inhibitory control) and facilitating strategies (i.e., activation control) for reducing the impact of stressors (Smeijers et al., 2020). Correspondingly, children’s knowledge of the nature, causes, and meaning of emotions may increase their understanding of others’ mental states, beliefs, and actions and, in turn, increase their emotion regulation and mental health (Wu et al., 2021). According to social information processing theory (e.g., Smeijers et al., 2020), effortful control reduces children’s risk for psychopathology by inhibiting the reflexive generation of hostile attributions and ineffective solutions to social transgressions. Through a similar social information processing mechanism, emotion knowledge is posited to decrease malevolent interpretations of others’ actions, facilitate social goals, and increase the formulation and enactment of constructive solutions that reduce their vulnerability to emotional and behavioral problems (Choe et al., 2013; Smeijers et al., 2020).

Research supports the viability of children’s emotion understanding and effortful control as predictors of lower levels of their psychological problems. For example, children’s emotion knowledge is modestly associated with their internalizing and externalizing symptoms in meta-analyses and prospective longitudinal studies (e.g., Sadri & Yates, 2025; Trentacosta & Fine, 2010; Zhang et al., 2024). Meta-analyses have also shown that childhood effortful control is concurrently and prospectively associated with lower externalizing symptoms (Robson et al., 2020). In highlighting possible selectivity in the sequelae of effortful control, the findings from these meta-analyses were weaker and, in some cases, non-significant for internalizing symptoms. Thus, the research underscores the value testing cascade models of the emotion processing dimensions (i.e., effortful control, emotion understanding) as mediators in quadratic associations between parenting adversity and children’s psychological problems. Yet, at the same time, they also raise the possibility that effortful control and emotion understanding may operate in domain-specific, rather than domain-general, ways in predicting internalizing and externalizing symptoms.

### State of the empirical literature on steeling and hormesis in family adversity cascades

Research on steeling or hormesis effects has predominantly focused on testing children’s exposure to socialization adversity as a quadratic predictor of their internalizing and externalizing symptoms (e.g., Hidalgo et al., 2023; Meunier et al., 2012; Oshri et al., 2024). Findings from these studies have yielded mixed support for steeling effects. For example, cross-sectional curvilinear relations between parenting difficulties and youth internalizing and externalizing symptoms were significant in supporting steeling effects for single-informant but not cross-informant reports of parenting and child outcomes (Hidalgo et al., 2023). Likewise, in another study (Arbel et al., 2020), a signfiicant concurrent curvilinear link between parenting stress and adolescent internalizing symptoms corresponded with a steeling or hormesis pattern whereby youth exhibited the lowest emotional distress at moderate levels of parenting stress. However, prospective analyses did not replicate these findings. As another example, longitudinal analyses yielded some support for a curvilinear relationship between family risk factors and youth internalizing and externalizing symptoms, but the relation was cubic rather than quadratic in form (Oshri et al., 2024). Even less is known about whether steeling effects are expressed in emotion processing (Morris et al., 2007; Repetti et al., 2002; 2011). As the only study supporting steeling effects in the broad domain of emotion processing, Davies and colleagues (2022) showed that children exhibited the lowest distress responses to interparental conflict when they witnessed moderate levels of interparental conflict.

### The present study

Our goal of testing steeling or hormesis effects models of parenting adversity in developmental cascades was designed to address significant gaps in the literature. Conceptual models have proposed that exposure to moderate family adversity may strengthen children’s emotional proficiencies and, in turn, inoculate them from psychopathology (Liu, 2015; Oshri, 2023; Repetti & Robles, 2016; Seery, 2011). However, no studies have examined whether experiencing moderate parenting adversity buffers children from psychological problems through their association with strength-based emotional competencies. Therefore, our paper breaks new ground by testing emotional competencies of children’s effortful control and emotion knowledge as proximal mediators in relations between a quadratic index of their exposure to parenting adversity and their internalizing and externalizing symptoms.

We aimed to examine steeling effects in children as they transition to the early elementary school. Although prior studies have predominantly relied on adolescent and adult samples in tests of family adversity as a quadratic predictor (Arbel et al., 2020; Hidalgo et al., 2023; Oshri et al., 2024; Seery, 2011), there are strong bases for hypothesizing that steeling or hormesis effects operating through effortful control and emotion understanding may be pronounced during the entry to formal schooling. According to developmental models (e.g., Del Giudice & Belsky, 2011; Koss & Gunnar, 2018), children experience heightened sensitivity to family experiences that are translated into stable adaptational patterns and adjustment during the transition from early to middle childhood. As two key dimensions of adaptation, children’s emotion knowledge and effortful control exhibit heightened plasticity based on the quality of their socialization histories during this developmental period (e.g., Berzenski & Yates, 2017; Neppl et al., 2020). Finally, research has documented that individual differences in children’s emotion understanding and effortful control during the formal school transition are precursors of their mental health (e.g., Fine et al., 2003; Robson et al., 2020). Thus, there is ample support for expecting that steeling experiences of parenting adversity may operate through emotion knowledge and effortful control mechanisms for children during the transition to the early elementary school years.

To minimize the operation of mono-method and mono-informant bias, we implemented a multi-method (i.e., observations, q-sort ratings, structured tests, surveys), multi-informant (i.e., trained observers, experimenters, children, teachers, parents) measurement battery in a longitudinal design with three annual measurement occasions the year before children enrolled in kindergarten. Based on recommendations for advancing a new generation of research on steeling effects (Liu, 2015), we examined whether the quadratic index of parenting adversity at Wave 1 was a significant predictor of children’s emotion knowledge and effortful control at Wave 2 while specifying the linear parenting adversity variable and potential confounding variables (i.e., household income; child age, sex, and psychological symptoms) at Wave 1 as simultaneous predictors. To model the second hypothesized links in the cascade, children’s emotion knowledge and effortful control at Wave 2, in turn, were specified as predictors of lower levels of their internalizing and externalizing symptoms at Wave 3. In addition, we employed a fully lagged moderated-mediation model by controlling for Wave 1 measures of the two proposed mediators and outcomes. In characterizing the form of curvilinear effects, we used the latest follow-up analyses to precisely identify the strengthening, buffering, and toxic zones of steeling findings for children in our sample (Oshri et al., 2024).

Guided by steeling and hormesis theory and research (Hidalgo et al., 2023; Oshri, 2023; Repetti & Robles, 2016; Rutter, 2012), we hypothesized that the quadratic index of parenting adversity exposure would significantly predict children’s subsequent emotion knowledge and effortful control. Based on the conceptualization of steeling or hormesis zones (Oshri et al., 2024; Oshri, 2023), we further hypothesized that children’s emotion knowledge and effortful control would be relatively equally represented across the strengthening, buffering, and toxic zones of family adversity. In examining the second part of the moderated-mediation cascade, we predicted that children’s emotion knowledge and effortful control would prospectively predict their diminished risk for internalizing and externalizing symptoms. To examine generalizability and specificity in these associations, our analytic plan tested whether pathways between children’s two dimensions of emotion processing and their psychological symptoms varied significantly as a function of the domain of psychological problems. Given the paucity of prior research testing the selectivity of the sequelae of children’s emotion understanding, we did not formulate hypotheses on its role as a domain-specific or domain-general predictor of psychological symptoms. However, based on prior meta-analytic and longitudinal research (Robson et al., 2020), we proposed that effortful control would be a significantly stronger predictor of children’s lower externalizing symptoms than their internalizing symptoms.

## Method

### Participants

Participants included 238 mothers and preschool children from a moderate-sized metropolitan area and surrounding county in the Northeastern United States. We recruited through multiple agencies, including pre-K programs, Head Start agencies, and public and private childcare providers. The average ages of children at Waves 1, 2, and 3 were 4.38 years (*SD* = .32), 5.61 years (*SD* = .41), and 6.59 years (*SD* = .40), respectively, with 52% of the sample consisting of female children. The median household income of the families was $72,000 per year (range = $500 – $260,000), with 32% of families receiving public assistance. Parent educational attainment was as follows: less than a high school diploma or equivalent (5%), high school diploma or equivalent (18%), some college (23%), bachelor’s degree (28%), and graduate training (26%). Children were primarily White (68%; 61% non-Latinx White), followed by children who were Black or African American (18%), multiracial (10%), or another race (4%). Approximately 16% of children were Latinx. At Wave 1, 100% of the mothers were biological parents. Mothers, partners, and children lived together in the same household for at least 10 months, and all had regular contact as a triad over the past year (i.e., at least 2 to 3 times per week across the prior year). Most mothers were married (73%), with the remaining couples in long-term committed relationships (20%), engaged to be married (5%), or in dating relationships (2%). Retention across the three annual measurement occasions was 88%. Data collection took place between 2018 and 2022.

### Procedures and measures

Parents and children participated in visits to a research center laboratory at the three waves of data collection. The number of families participating in data collection at Waves 2 and 3 was 201 and 210, respectively. The Institutional Review Board at the University of Rochester approved the research procedures under the title “Family Relationships and Children’s Functioning” prior to conducting the study (Approval # 00070387). Teachers completed surveys at Waves 1 and 3. Families were compensated for their participation ($225 for 2 visits at Wave 1; $250 for 2 visits at Wave 2; $200 for 1 visit at Wave 3), and children received small toys at each visit. Teachers who participated received $30 at each wave. Teachers provided reports for 84% (*n* = 199) and 93% (*n* = 194) of the children who participated in data collection at Waves 1 and 3, respectively.

#### Parenting adversity

At Wave 1, parents and children participated in three family interaction tasks that were video recorded for subsequent coding. Each coding team consisted of a primary coder and a reliability coder who independently coded at least 20% of the videos. For the first task, mothers, partners, and children were asked to work together for 6 minutes to build a tower out of blocks that exceeded the height of a very difficult-to-achieve record tower. No further instructions or structure were provided to maximize the likelihood that parents would adopt characteristic ways of interacting with their children. Different primary coders separately rated maternal and partner parenting behaviors along five 9-point scales (1 = *Not at all characteristic*; 9 = *Mainly characteristic*) that were adapted from the Iowa Family Interactions Scales (IFIRS; Melby & Conger, 2001). Codes included: (1) Intrusiveness, parental behaviors that interrupt and control their children’s activities, constrain their autonomy, and limit their self-initiated exploration; (2) Disengagement, parental displays of apathy, indifference, and detachment that minimize contact with the child; (3) Sensitivity, parental responsive behaviors to their children’s needs, emotional states, and abilities; (4) Positive Reinforcement, parental use of praise, approvals, affection and rewards in response to their children’s positive behaviors; (5) Autonomy Support, parental encouragement of their children’s initiatives, demonstrations of competence, and abilities to solve problems and make decisions commensurate with their developmental level. Interrater reliability, indexed by intraclass correlation coefficients (ICCs), ranged from .91 to .97 across the 10 codes.

For the remaining two tasks at Wave 1, mothers and partners participated in a parent–child problem-solving task with the child. Mothers and partners completed the task separately with their children at different visits to the lab spaced within one week of each other. Parents selected one discipline topic that they felt comfortable discussing with their child (e.g., listening to parents, picking up toys, fighting with siblings, bedtime routines, etc.) before the beginning of the task. Parents then discussed the topic with their children for 6 min. after they entered the room. Coders rated three adapted IFIRS codes for mothers and partners separately along 9-point scales (1 = *Not at all characteristic*; 9 = *Mainly characteristic*) (Melby & Conger, 2001). Codes included: (1) Psychological Control, characterized by parental disregard of their children’s perspectives and psychological domination of the interaction in ways that preclude children from functioning independently; (2) Inductive Reasoning, comprised of parental efforts to promote child processing and internalization of the consequences of their behaviors and the perspectives of others through explanations and discussions that are neutral or positive in valence; and (3) Sensitivity, indexed by parental abilities to accurately respond to children’s moods, cognitive states, and needs. *ICC*s, indexing interrater reliability, ranged from .79 to .94 across the 6 codes. To obtain a comprehensive and parsimonious composite of parenting adversity, we calculated the mean of the 16 codes from the three interaction tasks after reverse scoring the 10 supportive codes so that higher scores reflected greater adversity (*α* = .81).

#### Children’s emotion knowledge

To assess children’s emotion knowledge at Waves 1 and 2, children completed emotion recognition and emotion situation knowledge tasks. The emotion recognition measure consisted of an abbreviated version of the Morphed Faces Task to assess children’s abilities to recognize facial expressions of emotions across a spectrum of different intensities (Gibb et al., 2009). Relative to the use of prototypical emotional expressions in other assessments, the presentation of emotions of varying intensities in the Morphed Faces Task was designed to represent more complex, diverse emotional displays that occur in the daily lives of children. The task stimuli consisted of portrayals of happy, sad, and angry facial expressions of emotion depicted by two female and male actors from a standardized stimulus set (see Gibb et al., 2009). Emotion expressions of varying intensity were created by using computer software to morph the neutral facial expression of the actor with happy, sad, and angry facial expressions of the same actor in increments of 10, 30, 40, 60, 70%, and 90%. Synthesizing the pure expression of the target emotion with the neutral expression generated systematic variations in the intensity of happy, sad, and angry displays (e.g., 90% neutral with 10% angry; 40% neutral with 60% angry, etc.). The resulting task consisted of 72 trails (i.e., 6 morphs × 3 emotions × 4 actors). Each image was 26.5 cm tall and 16.5 cm wide and presented sequentially in the middle of the screen for a 3 s duration. After each trial, children responded verbally to the experimenter question: “Is this person happy, sad, or mad?” Emotion recognition scores at each wave consisted of the number of trials in which children correctly identified the emotion display.

We measured children’s emotion situation knowledge through the Emotions Subtest of the Schultz Test of Emotion Processing (STEP; Schultz et al., 2010). The STEP consisted of 18 videos of child actors in situations that prototypically elicit happiness, sadness, and fear (6 videos per emotion). After each video, the experimenter asked the child, “Does [the protagonist] feel happy, sad, angry, or scared?” For example, one of the videos for happiness involved a girl complimenting another girl (i.e., the protagonist) on her drawing. An emotion perspective-taking score was calculated at each wave as the proportion of correctly attributed emotions across the videos. At each wave, we created an overall composite of emotion knowledge by standardizing and averaging the two tasks. Our compositing was supported by the significant and moderate correlation between the two measures at Waves 1 (*r* = .40, *p* < .001) and 2 (*r* = .34, *p* < .001).

#### Children’s effortful control

At Waves 1 and 2, we used four assessments to capture children’s effortful control or their ability to organize reflective, purposeful, and internally guided responses to challenges. First, in the Gift Delay Task (Kochasnka & Knaack, 2003), children were instructed to stay seated and wait to open a bag containing a gift for them while an experimenter returned with a bow. After returning 3 min later, the experimenter invited the child to open the gift. Coders recorded the latency of each child to: (a) leave their seat, (b) touch the gift bag, (c) peer into the bag, and (d) put their hand in bag. Latencies were quantified on a scale ranging from 0 (i.e., child engaged in the action before the experimenter left the room) to 180 (child did not engage in the action) seconds. Longer latencies indicated greater effortful control (Kochanska & Knaack, 2003). Consistent with previous approaches (e.g., Spinrad et al., 2007), trained coders also rated children’s effortful control along a 9-point dimensional scale ranging from 1 (*No effortful control*) to 9 (*Intense effortful control*). Behaviors reflecting *no effortful control* included standing and peeking into the bag or touching the gift in the bag within one minute of being left alone during the delay period. Behaviors reflecting *intense effortful control* included the ability to inhibit impulses to move toward the bag, touch the bag, peak at the gift, or pull the gift out of the bag. Thus, high scores reflected the ability of children to successfully resist the temptation to open the gift (i.e., rewarding stimulus) while following the instructions to sit and wait until the experimenter returned before pulling the gift out the bag. *ICC*s, indexing two coders independent ratings on 100% of the videos, ranged from .94 to 1.00 across the latency and molar codes. We created an effortful control composite by standardizing and averaging the 5 codes (*α* = .87).

Second, children participated in an adapted version of the Lab-TAB Slides task (Goldsmith et al., 1999) to assess their ability to effortfully focus and sustain attention in a tedious activity. The Slides task consisted of the presentation of mundane pictures of children, children with parents, and nature scenes on a laptop screen. The task was organized around three blocks of five visual stimuli presented in a sequence where the duration of each stimulus presentation increased in 2 sec increments from 7 to 15 sec within each block. Children were instructed to look at the pictures during the task (total task duration: 2 min and 45 sec). To assess children’s ability to persist and sustain attention, trained coders rated children’s attentional focus on a 9-point dimensional scale. Low scores (1 = *Negligible attentional focus*) reflected children’s inability to concentrate for more than a few seconds at a time even with regular reminders of the experimenter. Conversely, high scores (9 = *High attentional focus*) reflected a high capacity to concentrate on the images throughout the task, with minimal (i.e., a total of a few seconds at most) or no off-task behavior. Interrater reliability based on two coders rating over 60% of the videos at each wave were good (*ICC* = .94 and .87 at Waves 1 and 2, respectively).

For the final two indicators of effortful control, the pair of experimenters overseeing child data collection activities completed the California Child Q-set (CCQ; Block, 2008). The “child” experimenter organized all the child’s activities throughout the visits, whereas the other “video” experimenter videotaped the tasks involving the child during visits. The CCQ requires raters to sort 100 descriptors of children’s behavior (e.g., “Is shy and reserved, makes social contacts slowly”) into nine different piles ranging from (1) “extremely uncharacteristic” to (9) “extremely characteristic.” Experimenters received 6 to 7 hours of CCQ training through instructional lessons, shadowing expert-led visits, completing supervised Q-sorts, and reviewing videotaped sessions of child activities from a prior study. To complete the Q-sort assessment, experimenters took notes on child behaviors during and after each visit. Ratings were based on the “child” and “video” experimenters observing child behavior for approximately 5 hours and 3.5 hours, respectively. Effortful control was assessed using the nine-item CCQ Conscientiousness scale (e.g., “Is persistent, does not give up easily,” “Is planful, thinks ahead”; *α* = .81) (John et al., 1994). Consistent with definition of effortful control, conscientiousness is defined as “socially prescribed impulse control that facilitates task and goal-directed behavior (John & Srivastava, 1999, p. 121).” The CCQ conscientiousness scale has been conceptualized and assessed as an indicator effortful control with children during early and middle childhood (e.g., Ahadi & Rothbart, 1994; Davies et al., 2025). The four indicators were standardized and averaged into an effortful control composite at Waves 1 (*α* = .69) and 2 (*α* = .70).

#### Children’s externalizing and internalizing symptoms

To assess children’s externalizing symptoms at Waves 1 and 3, teachers completed the Oppositional Defiant (9 items, “Has temper tantrums or hot temper”), Conduct Problems (11 items, “Lies or cheats”), and ADHD Symptoms (15 items, “Interrupts or butts in on others”) Scales from the MacArthur Health and Behavior Questionnaire (HBQ; Ablow et al., 1999). Response alternatives for the items were 0 = *Never or not true*, 1 = *Sometimes or somewhat true*, and 2 = *Often or very true*. Research supports the validity and reliability for these scales (e.g., Ablow et al., 1999). Internal consistencies (*α*) in this sample at Waves 1 and 3, respectively, were .90 and .89 for Oppositional Defiant, .90 and .85 for Conduct Problems, and .94 and .94 for ADHD. We averaged three measures together within each measurement occasion to create a composite of externalizing symptoms at Waves 1 (scale level *α* = .87) and 3 (scale level *α* = .76). To assess internalizing symptoms at Waves 1 and 3, teachers also completed the HBQ Overanxious (8 items; “Nervous, high-strung, or tense”), Depression (6 items; “Unhappy, sad, or depressed”), and Social Withdrawal (9 items; “Withdraws from peer activities”) Scales (Ablow et al., 1999). Internal consistencies (*α*) at Waves 1 and 3, respectively, were .76 and .82 for Anxiety, .72 and .79 for Depression, and .79 and .84 for Social Withdrawal Scales. We averaged the scales together to create measures of internalizing symptoms at Waves 1 (scale level *α* = .74) and 3 (scale level *α* = .77).

#### Demographic characteristics (Covariates)

Demographic covariates from a Wave 1 maternal interview consisted of (1) child sex (0 = female; 1 = male); (2) annual household income in units of $1000; and (3) child age in years.

## Results

Table 1 shows the means, standard deviations, and correlations for the variables included in the primary analyses. Parenting adversity at Wave 1 was moderately associated with lower levels of children’s effortful control and emotion knowledge at Waves 1 and 2. Wave 2 effortful control and emotion knowledge were also correlates of children’s lower internalizing and externalizing symptoms at Wave 3. Missing data for the variables in the primary analyses were minimal (13.7 %). Little’s test further supported the conclusion that the data were missing completely at random, *χ* ^*2*^ = 187.09, *df* = 186, *p* = .46 (Little, 1988). Consistent with recommendations, (Schlomer et al., 2010), we used full-information maximum likelihood data estimation methods to retain the full sample for all subsequent analyses. For all primary analyses, we conducted path analyses with Amos 29.0 software (Arbuckle, 2021).

**Table 1.** Means, standard deviations, and correlations among the primary variables in the study

	Mean	SD	1	2	3	4	5	6	7	8	9	10	11
Covariates													
1. Child Sex	--	--	--										
2. Child Age	4.38	0.32	.01	--									
3. Annual Income	80.35	49.12	.08	−.12	--								
Wave 1 Family Socialization													
4. Parenting Adversity	4.62	1.06	.03	.09	−.46*	--							
Wave 1 Child Mediators													
5. Effortful Control	0.00	0.68	−.03	.05	.25*	−.35*	--						
6. Emotion Understanding	−0.02	0.87	−.04	.24*	.29*	−.21*	.32*	--					
Wave 2 Child Mediators													
7. Effortful Control	0.00	0.68	−.06	.04	.27*	−.31*	.54*	.31*	--				
8. Emotion Understanding	0.01	0.83	−.09	.12	.25*	−.24*	.24*	.52*	.37*	--			
Wave 1 Child Psychological Adjustment													
9. Internalizing Symptoms	0.27	0.23	.04	.14	−.01	−.02	.04	.06	.00	−.08	--		
10. Externalizing Symptoms	0.25	0.32	.28*	.04	−.06	.12	−.19*	−.04	−.28*	−.12	.33*	--	
Wave 3 Child Psychological Adjustment													
11. Internalizing Symptoms	0.26	0.27	.02	.07	−.01	.01	.02	−.11	−.18*	−.29*	.17*	.07	--
12. Externalizing Symptoms	0.21	0.27	.20*	−.01	−.11	.16*	−.13	−.11	−.38*	−.24*	.06	.33*	.50*

*Note*. Annual Income is indexed in units of $1000. Child sex: 0 = female; 1 = male. * *p* < .05.

### Primary analyses: effortful control and emotion understanding as mediators

To test our steeling effects hypotheses, our path model specified the linear and quadratic parenting adversity variables at Wave 1 as predictors of (a) the proposed mediators of children’s effortful control and emotion knowledge at Wave 2 and (b) their internalizing and externalizing symptoms at Wave 3. The linear composite of parenting adversity was centered prior to creating the quadratic parenting adversity term. In turn, we estimated structural paths between each of proposed mediators of children’s effortful control and emotion knowledge at Wave 2 and their internalizing and externalizing symptoms at Wave 3. The three demographic covariates (i.e., child sex and age, household income) were also estimated as predictors of the two proposed child mediators at Wave 2 and two child sequelae at Wave 3. To increase analytic rigor, we specified autoregressive paths using Wave 1 measures of the mediators and child psychological symptoms. To account for the possibility that steeling effects were artifacts of concurrent links among parenting adversity and children’s psychological outcomes, we also specified structural paths between the two forms of psychological symptoms at Wave 1 and the children’s effortful control and emotion knowledge at Wave 2. Finally, although not shown in the figure for clarity, we estimated correlations between: (1) all pairs of exogenous variables; (2) the residuals for the two mediators at Wave 2; and (3) the residuals for the two child outcomes at Wave 3.

**Figure 2.**
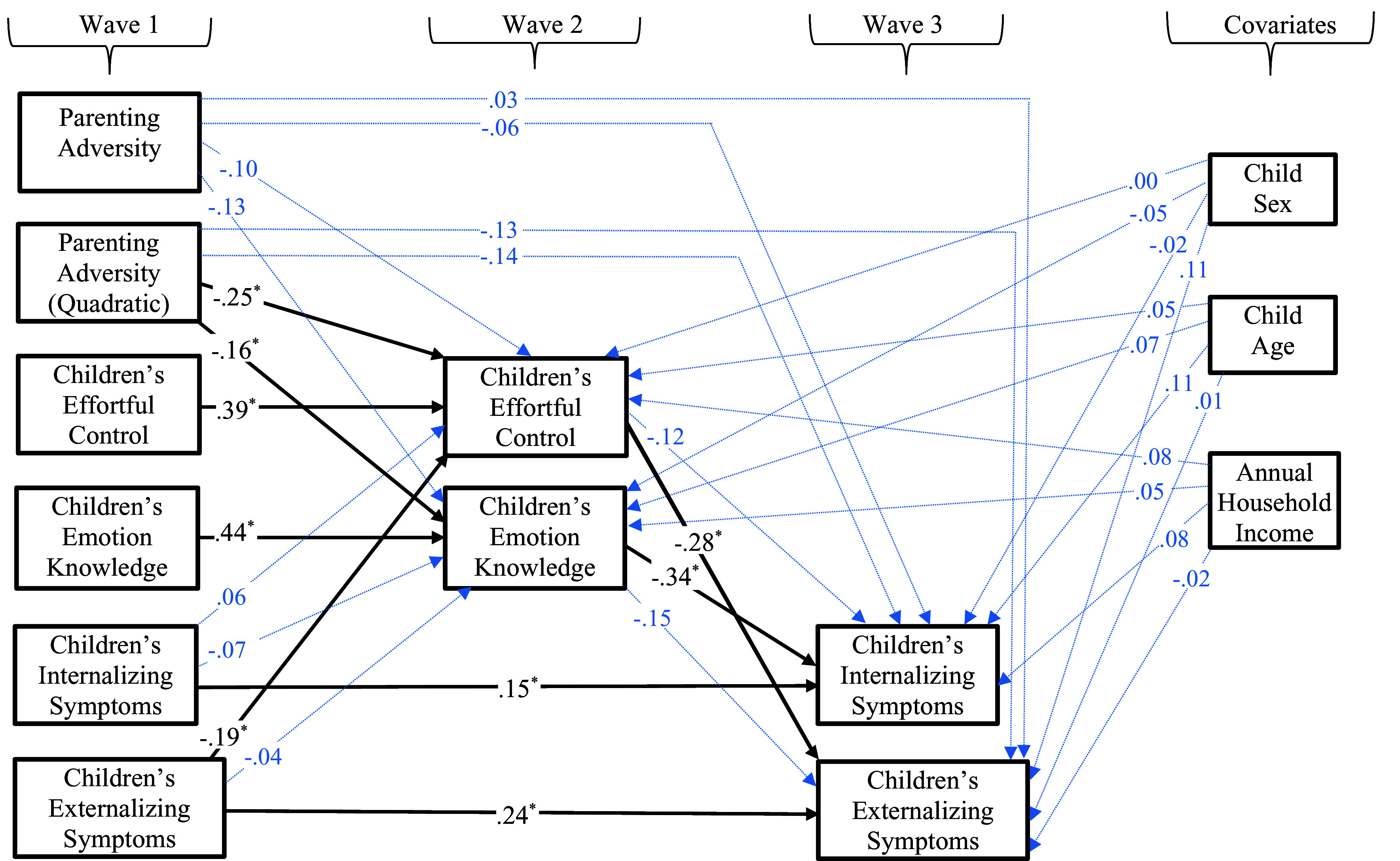
Results of the prospective path model testing effortful control and emotion knowledge as mediators in the quadratic association between parenting adversity and children’s internalizing and externalizing symptoms. * *p* < .05.

As depicted in Figure 2, the final model provided a good representation of the data, *χ* ^*2*^(8) = 7.52, *p* = .48, *CFI* = 1.00, *RMSEA* = .00. Autoregressive paths for all the endogenous variables were significant: children’s effortful control, *β* = .39, *p* < .001; emotion knowledge, *β* = .44, *p* < .001; internalizing symptoms, *β* = .15, *p* = .02; and externalizing symptoms, *β* = .24, *p* < .001. In addition, children’s externalizing symptoms at Wave 1 predicted their lower levels of effortful control at Wave 2, *β* = −.19, *p* = .009. Although the bivariate correlations in Table 1 indicated that parenting adversity was significantly correlated with lower levels of Wave 2 effortful control and emotion understanding and higher externalizing symptoms at Wave 3, it was not a significant predictor of the proposed mediators or outcomes in the broader autoregressive model that also contained the quadratic adversity term or covariates. In addition, the direct paths between quadratic predictor of parenting adversity and children’s internalizing and externalizing were not significant (see Figure 2).

#### Parenting adversity as a curvilinear predictor of an effortful control cascade

Turning to structural paths relevant to our study aims, the Wave 1 quadratic variable of parenting adversity significantly predicted children’s effortful control at Wave 2, *β* = −.25, *p* < .001. Wave 2 effortful control, in turn, predicted lower child externalizing symptoms, *β* = −.28, *p* < .001, but not internalizing symptoms, at Wave 3, *β* = −.12, *p* = .15. Parameter comparison analyses indicated that effortful control was a stronger predictor of children’s externalizing symptoms than their internalizing symptoms, *z* = 1.98, *p* < .05. Supporting moderated mediation, asymmetrical confidence interval analyses revealed that the indirect path involving Wave 1 quadratic family adversity, Wave 2 child effortful control, and Wave 3 child externalizing symptoms was significant, *b* = .013, *CI* [.004, .025] (MacKinnon et al., 2007).

**Figure 3.**
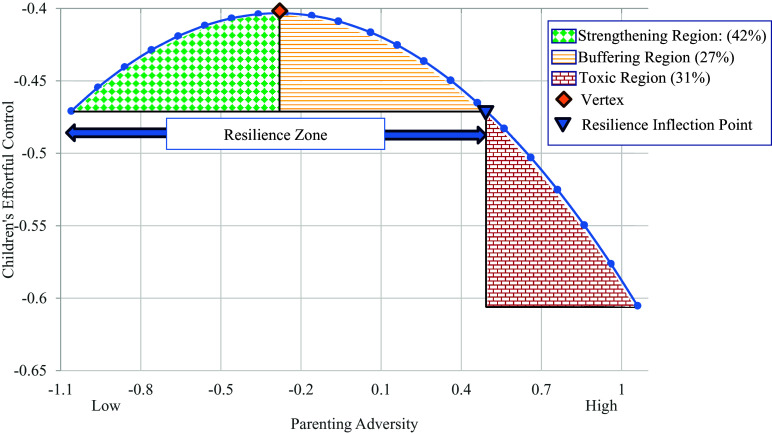
The graphical plot depicting the quadratic relation between exposure to parenting adversity and children’s effortful control.

To characterize the quadratic relation between parenting adversity and children’s effortful control, we calculated the curvilinear graphical plot across the range of ± 1 *SD* of the mean of family adversity. The resulting plot depicted in Figure 3 resembled an inverted J-shaped function. We calculated the strengthening, buffering, and toxic regions of the quadratic association based on previously established procedures (e.g., Oshri et al., 2024). The vertex, indexing the point at which the parabolic function shifts direction, occurred at a value of −.27 (.26 *SD* below the *Mean*) on the centered parenting adversity variable. In the strengthening (i.e., green diamond grid pattern) region of the plot to the left of the vertex, the prospective relation between parenting adversity and children’s effortful control was positive when the average raw score of family adversity was below 4.35, a value that approaches the descriptive anchor (5) “somewhat characteristic” on the dimensional scales of the adversity codes. The percentage of children falling in this strengthening region of the plot was 42%. In the buffering area of the curve to the immediate right of the vertex, children no longer accrued benefits from progressively higher exposure to parenting adversity but still exhibited comparable or better effortful control relative to children exposed to minimal levels of adversity (i.e., −1 *SD* below the mean). As denoted by the downward swing in the curve, parenting adversity shifted to predict subsequent decreases in children’s effortful control as adversity increases from modest (raw *M* = 4.35, approaching “somewhat characteristic) to moderate (*M* = 5.11; “moderately characteristic”) levels. Approximately 27% of children in our sample fell within the buffering region of the curve. Finally, in the region of the plot to the right of the resilience inflection point, the toxic zone denotes the region where levels of adversity are associated with poorer effortful control in comparison to children who experienced minimal family adversity (−1 *SD*). As exposure to parenting adversity exceeded the moderately characteristic range, children exhibited subsequently poorer effort control, with 31% of the sample falling in the toxic region of the curve.

In addition, we conducted *regions of significance (RoS)* analyses to identify the areas along the parenting adversity variable where it significantly predicted children’s effortful control. To maintain consistency with the multivariate specifications in the primary analyses, our first step was to save unstandardized residual values for Wave 2 emotion understanding after regressing it onto the Wave 1 covariates (i.e., household income, autoregressive path for child effortful control, and child internalizing symptoms, externalizing problems, age, and sex) using Mplus 8.11 software (Muthén,& Muthén, 2023). As our next step, we used R Core Team, 2025) to bootstrap 1000 samples by random sampling with replacement based on data from the full sample. For each sample, we then fit a quadratic regression (Y ∼ b_0_+ b_1_*X + b_2_* X^2^) using Lavaan 0.6-19 (Rosseel, 2012) with FIML to retain the full sample, and we retained the regression coefficient estimates for b_1_ and b_2_ within each bootstrapped sample. To obtain the slope estimates at different levels of X (i.e., change in Y per unit change in X), we defined X-grid as the minimum–maximum value of X with 100 Xs in total. We then calculated the simple slopes of the quadratic function based on the Dy/Dx derivative = b_1_ + 2b_2_*X. The mean slope was derived from the 1000 bootstrapped samples with confident intervals of the slopes defined by 2.5 and 97.5 percentiles of the Dy/Dx derivative = b_1_ + 2b_2_*X. Slopes were significant if the entire 95% CI did not contain zero. Findings indicated that the *RoS* was significant at both the low and high ends of the parenting adversity variable. Parenting adversity specifically predicted subsequently lower levels of effortful control for children exposed to adversity levels above the value of −.25 (i.e., > −.24 *SD* below the *Mean;* raw value = 4.38), which was applicable for 57% of the children in our sample. Conversely, parenting adversity significantly predicted increases in effortful control for a smaller proportion (i.e., 6%) of children who were exposed to levels of adversity below −1.64 (< −1.55 *SD* below the *Mean*).

#### Parenting adversity as a curvilinear predictor of an emotion knowledge cascade

Results from the primary analyses in Figure 1 also indicated that the Wave 1 quadratic parenting adversity variable was a significant predictor of children’s emotion knowledge at Wave 2, *β* = −.16, *p* < .01. Children’s emotion knowledge at Wave 2, in turn, predicted lower levels of children’s internalizing symptoms, *β* = −.34, *p* < .001, but not their externalizing symptoms, *β* = −.15, *p* = .051. Pairwise parameter comparison tests further revealed that children’s emotion knowledge more strongly predicted decreases in their internalizing symptoms than their externalizing symptoms, *z* = 2.25, *p* = .02. Supporting mediational paths, asymmetrical confidence interval analyses indicated that the indirect path for the curvilinear parenting adversity composite at Wave 1, children’s emotion knowledge at Wave 2, and their Wave 3 internalizing symptoms was significant, *b* = .009, 95% *CI* [.002, .020] (MacKinnon et al., 2007).

**Figure 4.**
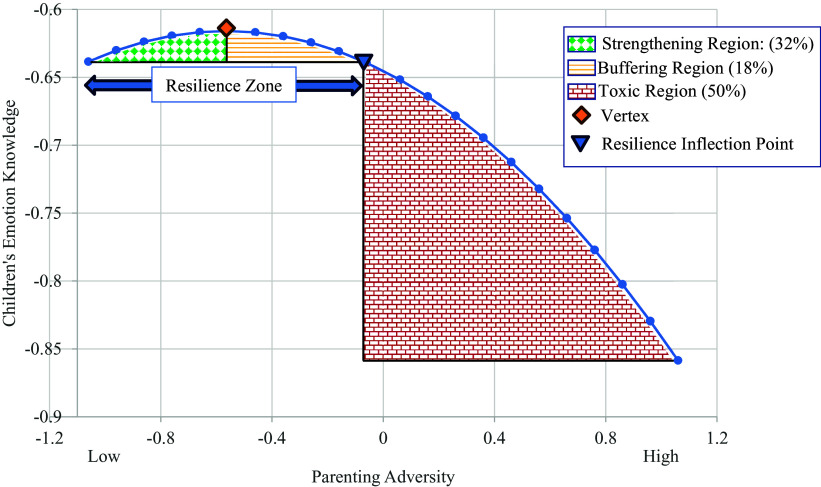
The graphical plot depicting the quadratic relation between exposure to parenting adversity and children’s emotion knowledge.

The graphical plot in Figure 4 depicts the shape of the curvilinear relation between parenting adversity and children’s emotion understanding within the bounds of ± 1 *SD* from the mean of parenting adversity. The form of the curvilinear slope followed an inverted J-shaped function that was consistent with steeling effects. There was a modest, positive association between parenting adversity and children’s emotion knowledge from the lowest regions of adversity exposure up to the vertex value of −.56 (.53 *SD* below the mean) on the centered parenting adversity variable (raw *Mean* = 4.06 on parenting adversity composite). This strengthening region, which is denoted by the green diamond matrix area of the plot, was applicable for 32% of the children in our sample. The association shifts in valence for values above the vertex, reflecting that parenting adversity was negatively related to children’s emotion knowledge as adversity approaches and exceeds moderate levels. The buffering region denoting the children who no longer benefit from exposure to parenting adversity but exhibit comparable or better emotion knowledge relative to children exposed to minimal levels of adversity (i.e., −1 *SD* below the mean) occurred in the region between the vertex (raw *Mean* = 4.06) and the resilience inflection point (raw family adversity *Mean* = 4.55). Children in this zone consisted of 18% of the sample. The toxic region in Figure 4, which is located to the immediate right of the resilience inflection point, denoted the area of the plot where exposure levels of adversity predicted poorer emotion understanding relative to children who experienced low levels of parenting adversity (−1 *SD* below the *Mean*). Approximately 50% of the children fell within the toxic region of the graphical plot.

Procedures for calculating *RoS* for effortful control were also conducted for children’s emotion understanding. Consistent with effortful control findings, parenting adversity was a significant predictor of lower emotion understanding for children. More specifically, parenting adversity was a risk factor for children when values exceeded −.19 on the centered adversity variable (raw value = 4.43). Approximately 53% of the children in the sample fell within this vulnerability range. However, in contrast to the effortful control findings, parenting adversity did not significantly predict better emotion understanding for any of the children at the lower bound values of adversity exposure.

## Discussion

Guided by strength-based steeling and hormesis models of family adversity (Liu, 2015; Oshri, 2023; Repetti & Robles, 2016), our study tested the hypothesis that children’s exposure to moderate, manageable levels of parenting adversity predicts their better psychological adjustment by strengthening their effortful control and emotion knowledge capacities. Findings from our fully lagged, moderated mediation analyses supported the steeling and hormesis models. In the first mediational pathway, the quadratic index of parenting adversity at Wave 1 predicted children’s emotion knowledge and effortful control at Wave 2 after the inclusion of autoregressive paths, the linear family adversity composite, and child adjustment (i.e., internalizing and externalizing symptoms) and demographic (i.e., sex, age, income) factors as covariates. In reflecting the second part of the mediational cascade, children’s Wave 2 effortful control selectively predicted lower levels of their Wave 3 externalizing symptoms while their emotion knowledge at Wave 2 specifically predicted their lower internalizing symptoms at Wave 3.

Consistent with steeling and hormesis models, the inverted J-shaped function of family adversity exposure indicated that increases in parenting adversity from negligible to moderate levels were associated with greater effortful control and emotion knowledge for a substantial minority (32 to 42%) of children in our sample. Signified by the buffering region to the immediate left of the vertices in Figures 3 and 4, an appreciable portion of children (18 – 27%) also exhibited better emotion processing capacities than their counterparts from low parenting adversity backgrounds despite not experiencing adaptive improvements in their functioning as exposure to adversity increased. Finally, the extreme right “toxic” regions of the figures revealed that another substantial proportion of children (31 – 50%) exhibited substantially lower emotion knowledge and effortful control than their peers exposed to minimal levels of parenting adversity.

### Steeling effects in relations between parenting adversity and child emotion processing

Our findings indicated that parenting adversity evidenced specificity as a quadratic predictor of the emotion processing (i.e., emotion knowledge, effortful control) dimensions rather than the forms of psychopathology (i.e., internalizing and externalizing symptoms) (Repetti et al., 2002; 2011). These results are consistent with many prior studies that have produced mixed or null findings in testing curvilinear relations between family adversity and children’s psychopathology (Arbel et al., 2020; Hidalgo et al., 2023; Oshri et al., 2024). From a theoretical perspective, individual differences in children’s emotion processing have been conceptualized as more proximal intermediary mechanisms that develop from family stress and, over time, cumulatively increase psychopathology (Repetti et al., 2002; 2011). Although this proposal from the risky families framework was initially developed to explain how linear indices of family adversity might indirectly increase children’s risk for psychopathology, our curvilinear findings suggest that the operation of effortful control and emotion knowledge are intermediary mechanisms that are sensitive to both the toxic toll of high adversity exposure and the strengthening and protective nature of mild, occasional, and manageable doses of family stress.

These results raise the question of how and why children exhibit the highest levels of effortful control and emotion understanding in the face of mild and moderate adversity in socialization contexts. In addressing this question, conceptualizations have identified interrelated classes of explanatory mechanisms for steeling responses that center on arousal and regulation parameters. In the arousal domain, challenging family interactions that do not rise to the level of palpable harshness may evoke moderate emotional arousal that optimizes attention, processing, and regulatory efforts (Lisitsa et al., 2021). For example, emotion theory supports the notion that mild negative emotions that result from manageable socialization stresses may enhance learning capacities and, in turn, increase children’s emotion knowledge and regulatory skills (Liu, 2015). Building on socioemotional theory, psychobiological models propose that mild, intermittent parenting adversity promotes reflective processing and regulation by generating moderate levels of physiological (e.g., glucocorticoids, norepinephrine) arousal (Blair, 2010; Liu, 2015). In supporting our documentation of toxic zones in the quadratic function, high levels of parenting adversity may disrupt these physiological systems and prefrontal-limbic functioning resulting in impairments in children’s emotion knowledge and effortful control (Blair, 2010).

In the regulation domain, steeling models propose that mild, occasional, and brief episodes of parental unresponsiveness, disengagement, and intrusiveness may provide opportunities for children to understand the complexity of emotional experiences and develop persistence, inhibitory control, and attentional facets of effortful control (Repetti & Robles, 2016; Rutter, 2012). As part of this process, repeated exposure to mild, occasional child-rearing difficulties may afford children with time to downregulate negative emotional arousal and, over time, lessen intense emotional experiences through habituation (Repetti & Robles, 2016). By developing social understanding and regulatory skills in these contexts, children may further enhance their perceived coping efficacy and confidence to withstand subsequent challenges (Liu, 2015; Repetti & Robles, 2016; Seery, 2011). Within hormesis models (Oshri et al., 2024; Oshri, 2023), these regulatory steeling effects are further instantiated in biological processes. Although the biological underpinnings are not well understood, preconditioning responses are theorized to be expressed in physiological and neural adaptations to mild stress that enhance child capacities to process and respond to future environmental challenges (Liu, 2015; Oshri, 2023). However, as the intensity and frequency of adversity increases to moderate or high levels, opportunities to “practice” processing and regulating environmental stressors in safe contexts may progressively diminish (Oshri, 2023; Repetti & Robles, 2016). In accord with this thesis, our findings indicated that emotion knowledge and effortful control capacities decreased significantly for children as their exposure to parenting difficulties increased from moderate to high levels.

### Developmental cascade from child emotion processing to psychological difficulties

In the second part of the steeling or hormesis cascade, the results supported specificity in paths between children’s emotion processing and their internalizing and externalizing symptoms. First, children’s effortful control at Wave 2 was a robust and stronger predictor of their Wave 3 externalizing symptoms relative to its null relations with Wave 3 internalizing symptoms. Selectivity in these findings is consistent with a recent meta-analytic results indicating that preschooler self-regulation was moderately related to their subsequent externalizing symptoms (*r* = −.30) and only weakly associated with their later internalizing symptoms (*r* = −.15) (Robson et al., 2020). In offering an explanation for these results, the response modulation model posits that effortful control parameters of attentional deployment (i.e., attention shifting and focusing), inhibitory control (i.e., inhibiting impulses), and activation control (i.e., deliberate generation of behaviors tailored to the problem) promote children’s perspective taking, reframing, and flexible problem-solving strategies for reducing anger and aggression (Nigg, 2017). According to evocative social process models (e.g., Alamos et al., 2022), effortful control may also be a promotive factor for children’s behavioral competence (i.e., lower externalizing problems) by evoking high-quality teacher and peer interactions. Finally, interpreted in resource allocation models (e.g., Chang et al., 2021), effortful control may be part of the early emergence of a slow-life strategy designed to gradually accumulate physical and social resources through greater social cooperation, altruism, and prosocial behavior and lower externalizing symptoms.

Second, in complementing the effortful control results, our analyses revealed that Wave 2 emotion knowledge was a significantly stronger predictor of children’s Wave 3 internalizing symptoms relative to its negligible association with Wave 3 externalizing symptoms. These results are difficult to interpret in the context of inconsistent findings on relations between emotion knowledge and children’s internalizing and externalizing findings in prior research. For example, our findings correspond with some research findings showing that emotion knowledge dimensions are related to internalizing symptoms but not externalizing symptoms (e.g., Göbel et al., 2016; Miller et al., 2006). However, a meta-analysis and other prior studies have documented that emotion knowledge is a significant modest correlate of both internalizing and externalizing symptoms for children (e.g., Ştefan & Avram, 2021; Trentacosta & Fine, 2010). Thus, although caution is warranted in drawing conclusions about the specificity of emotion knowledge as a predictor, several explanations are viable if our findings are replicated. First, emotion knowledge may buffer children from internalizing symptoms by promoting their downregulation of negative emotion and successful utilization of the adaptive regulatory and motivational functions of different types of emotions (Izard et al., 2008). Supporting this interpretation, cross-sectional findings have shown that children’s emotion regulation abilities account for the significant association between emotion knowledge and internalizing symptoms (Di Maggio et al., 2016). From a social information processing perspective, emotion knowledge might also increase children’s benign interpretations of social events and active solutions to social problems that reduce social anxiety, withdrawal, and depression (Lopes et al., 2012). Finally, emotion knowledge impairments may elicit confusion, distress, and disorientation that, in turn, increase risk for internalizing symptoms (Denham et al., 2012).

Selectivity in the sequelae of children’s effortful control and emotion knowledge may also be a product of their mediational interplay. Emotion understanding is regarded to be beneficial for children’s psychological functioning by, in part, increasing their self-regulation capacities. Correspondingly, children’s self-regulation has been proffered to promote children’s mental health by facilitating their acquisition of social and emotional knowledge (Denham et al., 2012). Supporting this hypothesis, prior research has identified bidirectional associations between children’s emotion knowledge and dimensions of their effortful control during their transition to school (Farrell & Gilpin, 2021). In our study, concurrent and prospective associations between effortful control and emotion knowledge were significant and moderate in magnitude (see Table 1). Thus, it is possible that effortful control is an indirect predictor of children’s lower internalizing symptoms through its association with greater emotion knowledge. Likewise, our findings might reflect that children’s emotion knowledge reduces their risk for externalizing symptoms by increasing effortful control.

### Limitations and future directions

Discussion of several study limitations and future directions is critical to interpreting the results. First, although participants in our study were from relatively diverse socioeconomic backgrounds, caution should be exercised in generalizing the findings beyond our community sample. For example, in underscoring the likelihood of variability in hormesis regions across contexts, steeling effects may be reduced or offset in highly adverse rearing conditions and magnified in high-resource socialization contexts (Oshri, 2023). Second, although our observations of maternal and partner parenting across three child-rearing contexts were designed to provide a rigorous assessment of children’s exposure to family adversity, future research would benefit from expanding tests of quadratic models beyond parenting adversity (e.g., coparenting relationship quality). As another complementary direction to our broad assessment of parenting adversity, distinguishing between different dimensions (e.g., parental sensitivity to child social signals, inductive discipline) may generate different quadratic curves and strengthening, buffering, and toxic regions.

Third, despite our efforts to carefully test parenting adversity as a quadratic predictor in a fully lagged, moderated mediation design, our prospective approach does not rule out the operation of all confounding variables. For example, mild or moderate levels of parenting difficulties may co-occur with another family factor (e.g., parental emotional expressiveness) that is a more proximal, linear predictor of emotion knowledge or effortful control. Fourth, because steeling effects might also be expressed through other mechanisms beyond children’s effortful control and emotion knowledge, expanding the search for mediators (e.g., child stress-sensitive physiological functioning, self-efficacy, coping styles) is an important future direction. Fifth, in contrast to the effortful control findings, the association between family adversity and children’s emotion understanding was not significant at any of the lower bound values of adversity in the bootstrapping analyses. Thus, although the graphical plot in Figure 4 contains a strengthening region, caution should be exercised in interpreting it due to the small, nonsignificant size of the area. Finally, although our curvilinear tests of parenting adversity were specifically guided by the predominance of quadratic conceptualizations of family adversity in the developmental psychopathology literature (Davies et al., 2022; Hidalgo et al., 2023; Oshri, 2023; Repetti & Robles, 2016; Rutter, 2012), exploring moderators of steeling or hormesis effects and other nonlinear forms of family risk (e.g., cubic, S-shaped functions) are important future steps for research (e.g., Oshri et al., 2024).

## Summary

In conclusion, although several conceptual models have proposed that mild or moderate levels of parenting adversity strengthen children’s psychological functioning (Liu, 2015; Oshri, 2023; Repetti & Robles, 2016), little is known about how the steeling or hormesis process unfolds in developmental cascades. Guided by the synthesis of steeling and risky families models, our paper was designed to break new ground by testing the hypothesis that family adversity increases children’’s risk for internalizing and externalizing symptoms through its quadratic association with two emotion processing dimensions: emotion knowledge and effortful control. Our results showed that children’s emotion knowledge mediated the quadratic relation between parenting adversity and their internalizing symptoms, whereas their effortful control mediated in the quadratic link between parenting adversity and their externalizing symptoms. Mild to moderate levels of parenting adversity predicted the highest levels of children’’s emotion knowledge and effortful control. Conversely, children exhibited diminished emotion knowledge and effortful control as their exposure to parenting adversity increased from moderate to high levels. The findings highlight the importance of complementing deficit models of family adversity with strength-based models of steeling and hormesis effects. Although it is premature to offer specific translational recommendations without further research, our results have the potential to inform clinical and policy efforts if the findings are replicated. Results specifically suggest that different clinical and policy approaches may be needed to promote the welfare of children exposed to low and high levels of parenting adversity. Children who experience minimal parenting adversity may benefit most from educational and therapeutic resiliency programs that strengthen psychological capacities (e.g., impulse regulation, social information processing) through controlled exposure to challenges (Rutter, 2012). Conversely, trauma-informed clinical and policy initiatives may be optimal for improving the lives of children exposed to high parenting adversity (Liu, 2015).

## Data Availability

The data and code necessary to reproduce the analyses presented in the paper are available at https://osf.io/py6ac/. Due to the extensive nature of the measures used in this multi-method longitudinal study, specific measures and materials can be accessed through requests to the first author.

## Pre-registration statement

Due to the relatively exploratory nature of the research questions, the study was not preregistered.
